# Variations in leopard cat (*Prionailurus bengalensis*) skull morphology and body size: sexual and geographic influences

**DOI:** 10.7717/peerj.1309

**Published:** 2015-10-06

**Authors:** Fernando L. Sicuro, Luiz Flamarion B. Oliveira

**Affiliations:** 1 BioVasc—Departamento de Ciências Fisiológicas—IBRAG, Universidade do Estado do Rio de Janeiro, Rio de Janeiro, Brazil; 2 Departamento de Vertebrados—Setor de Mamíferos, Museu Nacional—Universidade Federal do Rio de Janeiro, Rio de Janeiro, Brazil

**Keywords:** Morphometrics, Sexual dimorphism, Geographic morphotypes, Skull morphology, Mutivariate statistics, Leopard cat

## Abstract

The leopard cat, *Prionailurus bengalensis* (Kerr, 1792), is one of the most widespread Asian cats, occurring in continental eastern and southeastern Asia. Since 1929, several studies have focused on the morphology, ecology, and taxonomy of leopard cats. Nevertheless, hitherto there has been no agreement on basic aspects of leopard cat biology, such as the presence or absence of sexual dimorphism, morphological skull and body differences between the eleven recognized subspecies, and the biogeography of the different morphotypes. Twenty measurements on 25 adult leopard cat skulls from different Asian localities were analyzed through univariate and multivariate statistical approaches. Skull and external body measurements from studies over the last 77 years were assembled and organized in two categories: *full data* and *summary data*. Most of this database comprises small samples, which have never been statistically tested and compared with each other. *Full data* sets were tested with univariate and multivariate statistical analyses; *summary data* sets (i.e., means, SDs, and ranges) were analyzed through suitable univariate approaches. The independent analyses of the data from these works confirmed our original results and improved the overview of sexual dimorphism and geographical morphological variation among subspecies. Continental leopard cats have larger skulls and body dimensions. Skulls of Indochinese morphotypes have broader and higher features than those of continental morphotypes, while individuals from the Sunda Islands have skulls with comparatively narrow and low profiles. Cranial sexual dimorphism is present in different degrees among subspecies. Most display subtle sex-related variations in a few skull features. However, in some cases, sexual dimorphism in skull morphology is absent, such as in *P. b. sumatranus* and *P. b. borneoensis*. External body measurement comparisons also indicate the low degree of sexual dimorphism. Apart from the gonads, the longer hind foot of male leopard cats is the main feature of sexual dimorphism among *P. b. bengalensis* (and probably among *P. b. horsfieldii* too). External body measurements also indicated the absence of sexual dimorphism among individuals of *P. b. borneoensis*. Inter-subspecific skull comparisons provided a morphometric basis for differentiating some subspecies. *Prionailurus b. horsfieldii* and *P. b. bengalensis* were distinguished only by a subtle difference in PM^4^ size, indicating that overall skull morphology does not appear to support their separate taxonomical status, in spite of the marked differences reported in their coat patterns. Geological events affecting the Sunda Shelf connection between the Sunda Islands and the mainland during the Last Glacial Maximum seem to have influenced directly the morphological pattern shown by leopard cat subspecies nowadays.

## Introduction

The leopard cat, *Prionailurus bengalensis* (Kerr, 1792), has a wide distribution in southern and southeastern continental Asia, as well as in the Sunda Islands and the Philippines. It is also found in more northerly regions such as the Amur basin, Korea and the Japanese islands (Sunquist & Sunquist, 2002). The species occurs in habitats ranging from tropical rainforest to temperate broadleaf and, marginally, coniferous forest, as well as shrub forest and successional grasslands (MacDonald & Loveridge, 2010).

The existence of isolated populations across a wide geographical distribution has resulted in different morphotypes, some of them recognized as subspecies. Leopard cats from the Amur, for instance, are reported to be larger than those from southeastern Asian and island populations (Guggisberg, 1975; Heptner & Sludskii, 1992). Wozencraft (2005) acknowledges eleven subspecies: *P. b. bengalensis*, *P. b. alleni*, *P. b. borneoensis*, *P. b. chinensis*, *P. b. euptilurus*, *P. b. heaneyi*, *P. b. horsfieldii*, *P. b. javanensis*, *P. b. rabori*, *P. b. sumatranus*, and *P. b. trevelyani*. The Iriomote cat remains *incertae sedis* as a subspecies (*P. b. iriomotensis*) or a distinct species (*P. iriomotensis*) since morphological and molecular analyses are not congruent (Masuda & Yoshida, 1995; Johnson et al., 1999; Leyhausen & Pfleiderer, 1999). The IUCN Red List (Sanderson et al., 2008) classified *P. bengalensis* as a “least concern” species. However, due to deforestation, habitat alteration, and the economic value of its exuberantly spotted pelage, some subspecies face a more worrying future, such as *P. b. rabori*, cited as “vulnerable” (Lorica, 2008) and *P. b. iriomotensis*, classed as “critically endangered” (Izawa, 2008).

Felids have complex social systems. Availability of resources and stable environments in which they can rear their young are the main factors that lead females to establish their home ranges. There are many specific variations in the way females share their area of occurrence and in the degree of overlap between their home ranges (Sunquist & Sunquist, 2002). Male cats’ distributions are a function of the females’ distributions, and hence vary considerably according to the species’ social system. The standard social pattern of cats is characterized by male competition, where dominant males have access to home ranges containing more females. With some remarkable exceptions such as lions, male cheetahs and, to some extent, Canadian lynxes, adult felids live alone most of the time (Guggisberg, 1975; Nowak, 1999; Ruggiero et al., 1999; Sunquist & Sunquist, 2002). Female felids spend part of their lifetime caring for their cubs, while male parental care is rarely seen among cat species. These different social roles of the sexes are reflected in the marked sexual dimorphism observed along the Felidae. Morphological differences between sexes are reported for several cat species (Pocock, 1939; Guggisberg, 1975; Heptner & Sludskii, 1992; Nowak, 1999; Sunquist & Sunquist, 2002), and marked sexual size and shape dimorphism is also present in the skulls of most species (Sicuro & Oliveira, 2011). However, some species seem to display a more subtle morphological difference between sexes and contradictory descriptions are sometimes found even in the same papers. The causes of this lack or, at least, low level of sexual dimorphism are diverse. It may be attributed to different specific ecological and/or evolutionary trends among felid species, but also to loose descriptions that fail to recognize morphological variation between males and females. This seems to be the case with the leopard cat (*P. bengalensis*). The literature on this species is contradictory when discussing sexual dimorphism (in both external and internal features). For instance, Dollinger (1982), describing general aspects of *P. bengalensis* in his CITES report, stated that there is no marked sexual dimorphism in this species. Allen (1929), based on the analysis of 24 specimens from several locations in China, pointed out that “males are larger, with larger markings than females, and in old age the skulls have a low sagittal crest formed by the union of the temporal ridges, whereas in none of the females seen does this ridge form” (page 10, 1st paragraph). The same author in his book *The Mammals of China and Mongolia* (Allen, 1938) mentioned that “skulls of adult males are little larger than those of females, but the difference is not very great” (page 461, 2nd paragraph). Characteristics of the Amur subspecies of leopard cat (*P. b. euptilurus*) were described by Heptner & Sludskii (1992). These authors remarked that “Sexual dimorphism is absent and age-related changes have not been described for Soviet forms” (page 334, 4th paragraph) but, a few lines below, Heptner & Sludskii (1992) asserted: “Sexual dimorphism is absent apart from somewhat smaller size of female skull” (page 335, 5th paragraph). The authors based their opinion on external and skull measurements of males and females, but no statistical tests of significance were presented.

Groves (1997) presented one of the most detailed studies of leopard cats in southeastern Asia in both peninsular and insular locations: Negros and Palawan in the Philippines; Sumatra, Java, Bali, and Borneo in Indonesia/Malaysia; and Mainland, corresponding to the Indochinese region. The author used 72 skulls (♂*n* = 42 and ♀*n* = 30), with from one to 12 individuals per location. His morphological analysis was based on three skull measurements (Greatest skull length, Condylobasal length, and Bizygomatic breadth), P^4^ length, external body measurements, and comparison of pelage patterns. He found pelage color and pattern differences between mainland specimens (Indochina) and insular/peninsular specimens (Sundaic subregion), with some differences in spots and stripes between individuals in this subregion depending on the island of origin. Groves (1997) also detected a decreasing gradient in the skull size of males in the order: Indochinese peninsula—Sumatra—Negros—Java—Borneo—Bali—Palawan, in a very complex biogeographical mosaic among these regions. Male and female skull size variation was also found in condylobasal length ratios. Depending on the location, female condylobasal length ranged from 90.1% to 98.6% of the males’ measurements (no statistical support was presented). Groves (1997) also performed a Discriminant Function Analysis (DFA) including adults and late-juveniles of both sexes, based on skull and P^4^ measurements, separated by location. The resulting plot showed a cluster of specimens from Borneo, Sumatra, and the Indochinese mainland, another of Javanese specimens, and two small clusters corresponding to the Philippines populations of Negros and Palawan. The author explained that “restricting the analysis to adults only reduced the dataset too much to achieve meaningful results” (Groves, 1997, page 333, end of 5th paragraph); even so, the clusters presented lie quite close to each other and no statistical significance of the DFA was presented. Groves (1997) concludes his paper with detailed diagnoses for *P. bengalensis* subspecies according to the locations studied.

Nowell & Jackson (1996), in their review of wild cats, cited the greater weight of males (*n* = 6) in relation to females (*n* = 2) as a parameter of sexual dimorphism among leopard cats. Nevertheless, Heptner & Sludskii (1992) pointed out that weight measurements on leopard cats are quite variable due to seasonal fluctuations. Izawa et al. (2009) also indicated weight and body length differences between males and females of two Japanese subspecies of leopard cat; however, no information about sample size was given.

On the other hand, descriptions of leopard cats’ sexual morphological differences are sometimes completely absent in comprehensive reviews of Felidae biology, evolution and conservation status, such as Guggisberg (1975), Sunquist & Sunquist (2002), and MacDonald & Loveridge (2010), as well as in the specific review on the species *P. bengalensis* by Yu & Wozencraft (in press), and in Habibi’s (2003) book about the mammals of Afghanistan. Pocock’s (1939) classic work made no direct reference to sexual dimorphism in three subspecies of leopard cat (*P. b. trevelyani*, *P. b. horsfieldii*, and *P. b. bengalensis*). The author did, however, present raw skull and external body measurements of individuals of both sexes of these subspecies, and concluded there was similarity of size among the three subspecies (Pocock, 1939). Furthermore, Sicuro & Oliveira (2011) in a broad morphological study on 34 extant felid species found no significant differences between male (*n* = 10) and female (*n* = 10) leopard cats, based on 20 skull measurements. However, the study addressed interspecific skull variations between felid species (rather than intraspecific variations), and the sample included specimens from different Asian locations (Sicuro & Oliveira, 2011).

The present study aims to present a new approach to skull and body variation in *P. bengalensis* according to sex and geographical origin. New morphometric data is presented and analyzed by univariate and multivariate methods. Data from earlier works are also revisited and reanalyzed. The main challenge is still the small sample sizes usually found in felid collections; therefore this study seeks to integrate morphometric data from the most representative studies on leopard cat morphology over the last 77 years, since Allen (1938).

## Material & Methods

Two types of data were analyzed: (1) original measurements taken directly in mammal collections and (2) data obtained from other authors’ publications. This second type of data was separated into two categories: *full data*, including the original measurements taken by different authors; and *summary data*, composed of means, standard deviations (SD), and *n*-values; all of these had also already been published, but at different times. These studies had attempted to document and analyze qualitative and quantitative variation in *P. bengalensis* subspecies. However, they often presented a limited statistical approach, appropriate at the time of their publication.

We took 20 measurements on 25 adult leopard cat skulls (♀*n* = 10, ♂*n* = 12, and no sex (n/s) identification *n* = 3) held in the Mammal Collection of the American Museum of Natural History (AMNH), New York, USA. The skull measurements were taken with a Mitutoyo 500–341 digital caliper (150 mm: 0.01 mm). The descriptions and acronyms of our measurements are listed in Table 1 and are based on Sicuro & Oliveira (2011). Contact the corresponding author for the raw data file.

**Table 1:** Skull measurements. Descriptions and acronyms of the skull measurements used in the morphometric analysis of the original data.

Skull measurement	Acronym	Description
Breadth of braincase	BBC	The widest point across parietals
Condylobasal length	CBL	From the anterior edge of the premaxillae to the most posterior projection of the occipital condyle
Condyle to canine length of jaw	CCL	From the posterior margin of the canine alveolus to the most posterior edge of the jaw condyle
Condyle to M_1_ length of jaw	CM_1_L	From the anterior margin of the M_1_ alveolus to the most posterior edge of the jaw condyle
Jaw height at M_1_	JHM_1_	Measured at mid-point of the dentary between M_1_ and P_4_
Jaw length	JL	From the anterior limit of the dentary bone between I_1_ to the posterior edge of the jaw condyle
Jaw width at M_1_	JWM_1_	Measured near the point of JHM_1_
Masseteric fossa length	MFL	From the lateral limit of the jaw condyle to the anterior limit of the masseteric fossa in the dentary
Masseteric moment arm	MMA	From the dorsal surface of the jaw condyle to the ventral border of the angular process
Masseteric scar length	MSL	Measured on the ventral side of zygomatic arch, from the anterior limit of muscle scar on the jugal to the anterior edge of the glenoid fossa (temporomandibular fossa)
Masseteric scar width	MSW	The widest part of the masseteric scar on the jugal bone
Mastoid breadth	MB	Greatest width of skull including the mastoids
Occipital height	OCH	From the ventral border of foramen magnum to the lowest limit of the middle of the complex muscle scar
Orbit to premaxilla length	OPL	From the anterior end of premaxilla to the anterior orbit rim
Postorbital constrictions	POC	The shortest distance across the top of the skull posterior to the postorbital process
Rostral width at the second premolar P^2^	RWP^2^	Width between external limits of maxillary bones about P^2^
Temporal fossa length	TFL	From the most posterior point of the temporal fossa to the supraorbital process
*Temporalis* muscle moment arm	TMA	From the posterior end of the condyle to the apex of the coronoid process
Tooth row length	TRL	from the anterior face of I^1^ to the posterior face of M^1^, both near the alveolus
Zygomatic arches internal breadth	ZIB	The greatest distance between the inner margins of the zygomatic arches

Due to the lack of larger subsamples in each subspecies, we separated the specimens into three major geographical domains: China (*n* = 10), Indochina (*n* = 5), and Sunda Islands (*n* = 9); one female specimen had no location determined. Four individuals in the Chinese subset came from Hainan Island. Individuals from Indochina came from India, Myanmar, Laos, and Thailand, while those in the Sunda Islands group came from Java, Sumatra, and Borneo.

The original measurements were log-_10_ transformed and tested for normality, homoscedasticity, kurtosis, and skewness. Based on the results of these tests, we chose between parametric (ANOVA and *t*-test) and non-parametric methods (Kruskal–Wallis ANOVA and Mann–Whitney tests) for univariate analyses. Principal Component Analysis (PCA) allowed for the interpretation of morphological skull variation among the leopard cat morphotypes from the major geographical domains. Discriminant Function Analysis (DFA) was used to improve our insight into sexual dimorphism and geographical variation in the skulls.

Literature data were obtained from Allen (1938), Pocock (1939), Tan (1984), Heptner & Sludskii (1992), Groves (1997), Lim (1999), Rajaratnam (2000), Sunquist & Sunquist (2002), and Grassman et al. (2005); these data were independently re-analyzed with univariate and multivariate methods and results were compared with the results of our own data. Statistical analyses of *full data* were performed with Statistica version 8.0 (StatSoft, 2007) and the analyses of *summary data* were performed with DeSummarize package (J Pezzullo, pers. comm, 2014) for the R Language and Environment for Statistical Computing version 3.0.2 (R Core Team, 2013).

Allen’s (1938) classic monograph *Mammals from China and Mongolia* provides a full table with nine skull measurements from 22 specimens of *P. b. chinensis* and one of *P. b. bengalensis*, most of them with sex identification. We used 20 specimens of Allen’s sample for univariate (Mann–Whitney test) and multivariate statistical analyses (DFA) to test for sexual dimorphism, and 22 individuals to assess subspecific morphological variation in the skulls. The single *P. b. bengalensis* was excluded from these analyses. Allen’s (1938) measurements used were: Greatest length, Basal length, Palatal length, Zygomatic width, Mastoid width, width across Molars, median length of Nasals, and Upper Cheek Teeth.

Pocock’s (1939) classic work, *Fauna of British India including Ceylon and Burma*, presents full data: nine skull measurements from 15 adult specimens of leopard cat, including *P. b. trevelyani* (*n* = 1, young adult), *P. b. horsfieldii* (*n* = 8), and *P. b. bengalensis* (*n* = 5, one male cited as young adult). Most of them were males (*n* = 10). There are also external body measurements of 11 specimens of these three subspecies (♂*n* = 8). We used non-parametric comparisons between sexes and subspecies (Mann–Whitney test).

Heptner & Sludskii (1992) presented summary data of skull and external body measurements of male and female leopard cats, including both adults and juvenile forms. There were eight skull measurements, but only six with a representative sample of individuals. Greatest length, Condylobasal length, Zygomatic width, Interorbital width, Postorbital width, and length of Upper Tooth Row with Canine were measured on adult males (*n* = 12) and subadult males (*n* = 8), and on adult females (*n* = 6) and subadult females (*n* = 12). We used the summary data to compare the sexual differences between adults and between subadults in separate *t*-tests. Heptner & Sludskii (1992) provided only means, sample sizes (*n*), and range values, but not standard deviations (SD). Since SD is needed for summary data analytical approaches, we used the Empirical Rule to estimate the SD from the range of each skull variable (Sternstein, 2005; Utts & Heckard, 2006). If one assumes the data have a normal frequency distribution, 68% of the values fall within one SD of the mean on both sides of the distribution, 95% of the values fall within two SD of the mean, and 99.7% of the values fall within three SD of the mean also on either side (considering a *z*-distribution). Therefore, we estimated the SD as being roughly equal to values falling between 1/4 and 1/6 of the range (Sternstein, 2005; Utts & Heckard, 2006). The means, SDs and *n*-values were then used to test skull sexual dimorphism in Amur leopard cats.

Groves (1997) presented three skull measurements and one of P^4^ summarized in a table with means, standard deviations, ranges and *n*-values. The sample was composed of leopard cats from Negros (♂*n* = 3 and ♀*n* = 2), Palawan (♂*n* = 5 and ♀*n* = 1), Mainland [Indochina] (♂*n* = 3 and ♀*n* = 6), Sumatra (♂*n* = 7 and ♀*n* = 4), Java (♂*n* = 12 and ♀*n* = 9), Borneo (♂*n* = 8 and ♀*n* = 7), and Bali (♂*n* = 4). We compared sexes and locations of the better sampled subgroups (*n* > 3) through *t*-tests and ANOVA on summary data. The DeSummarize ANOVA routine and the following Bonferroni post-hoc test were performed in R. Due to the small sample sizes and indirect assessment of these data, the results were regarded as trends for making inferences about sex and subspecific skull variation.

For external body measurements, Sunquist & Sunquist (2002) published a table summarizing head-and-body length, tail length, and weight data from eight studies of leopard cats from nine locations: China, Northern China, Southern China, India, Thailand, Sabah (Malaysian Borneo), Borneo, Russia, and Peninsular Malaysia. Three of those studies are listed as describing only one or two individuals or only male individuals (from Northern China, Southern China, Thailand and Borneo), and one presents only the range of the variable values, but not the means (Russia: Heptner & Sludskii, 1992). The individuals mentioned as coming from Southern China actually came from Eastern China, as reported by Tan (1984) in his original work. The four remaining studies are: Shaw, TH 1962 Economic fauna of China: Mammals, also found cited as: Shou Zhenghuang, 1962: *Monograph* of the *Economic Animals of China*: *Mammals* in Chinese (neither of the originals was consulted), Pocock (1939), Rajaratnam (2000) and Lim (1999). The original works of Pocock (1939), Lim (1999) and Rajaratnam (2000) presented tables with full data of external morphology, but no statistical analyses.

The material assembled by Pocock (1939) actually included different leopard cat subspecies (*P. b. trevelyani* ♂*n* = 1; *P. b. horsfieldii* ♂*n* = 2 and ♀*n* = 1; and *P. b. bengalensis* ♂*n* = 4 and ♀*n* = 2) from several locations (Pakistan, Northern, Southern and far Eastern India, Myanmar, and Nepal). Rajaratnam’s PhD thesis (2000) is one of the most comprehensive works on leopard cat ecology, in Tabin Wildlife Reserve in Sabah. External body measurements of five females (adult *n* = 3, subadult *n* = 2) and five males (adult *n* = 4, subadult *n* = 1) of *P. b. borneoensis* allowed for full data comparisons.

Heptner & Sludskii (1992) did not include mean values for the external body measurements of the adult females in their table, and therefore this group could not be assessed. On the other hand, this study presented information rarely found in works dealing with elusive small wild cats: a small, though representative, sample of subadult forms (aged up to one year) of the Amur leopard cat (♀*n* = 12, ♂*n* = 8). Although this was not the main aim of the present paper, these groups were analyzed by means of a *t*-test for summary data in order to shed some light on ontogenetic skull variation in *P. b. euptilurus*. Finally, Lim (1999) presented a list of 12 males and eight females of *P. b. bengalensis* from peninsular Malaysia.

In the present study, full external body measurement data provided by the authors cited above were statistically analyzed to test for morphological variation with sexual and taxonomic implications. Data from male leopard cats summarized by Sunquist & Sunquist (2002) were also compared across geographical regions through an ANOVA for summary data (R routine). Sunquist & Sunquist’s (2002) original table and values were checked and adjusted for the meta-analysis. As pointed out by Heptner & Sludskii (1992), weight measurements on leopard cats are quite variable due to seasonal fluctuations, thus this variable was not taken into account in our analyses.

Due to taxonomic controversies and the scarcity of material, the Iriomote cat, *P. b. iriomotensis*, was not included in this study.

## Results

### Analyses of original data

Samples of both male and female leopard cats were considered homoscedastic and without major distribution problems. However, due to the small sample sizes, non-parametric Mann–Whitney tests were used in the univariate comparisons. When the whole sample (all localities) was considered, only the Masseteric moment arm (MMA) was significantly different between males and females (Mann–Whitney *U*_2,22_ = 28.0, *p* < 0.04), denoting a greater distance between the jaw condyle and the angular process in males. In Chinese individuals (♀*n* = 3, ♂*n* = 5), significant differences were found between the sexes in mastoid breadth (MB), Zygomatic arches internal breadth (ZIB), Masseteric fossa length (MFL), and once again Masseter moment arm (MMA), indicating that male leopard cats have a slightly larger skull than females. However the significance of these results is *p* < 0.04 and, due to the small sample size, they should be regarded as trends.

**Figure 1:**
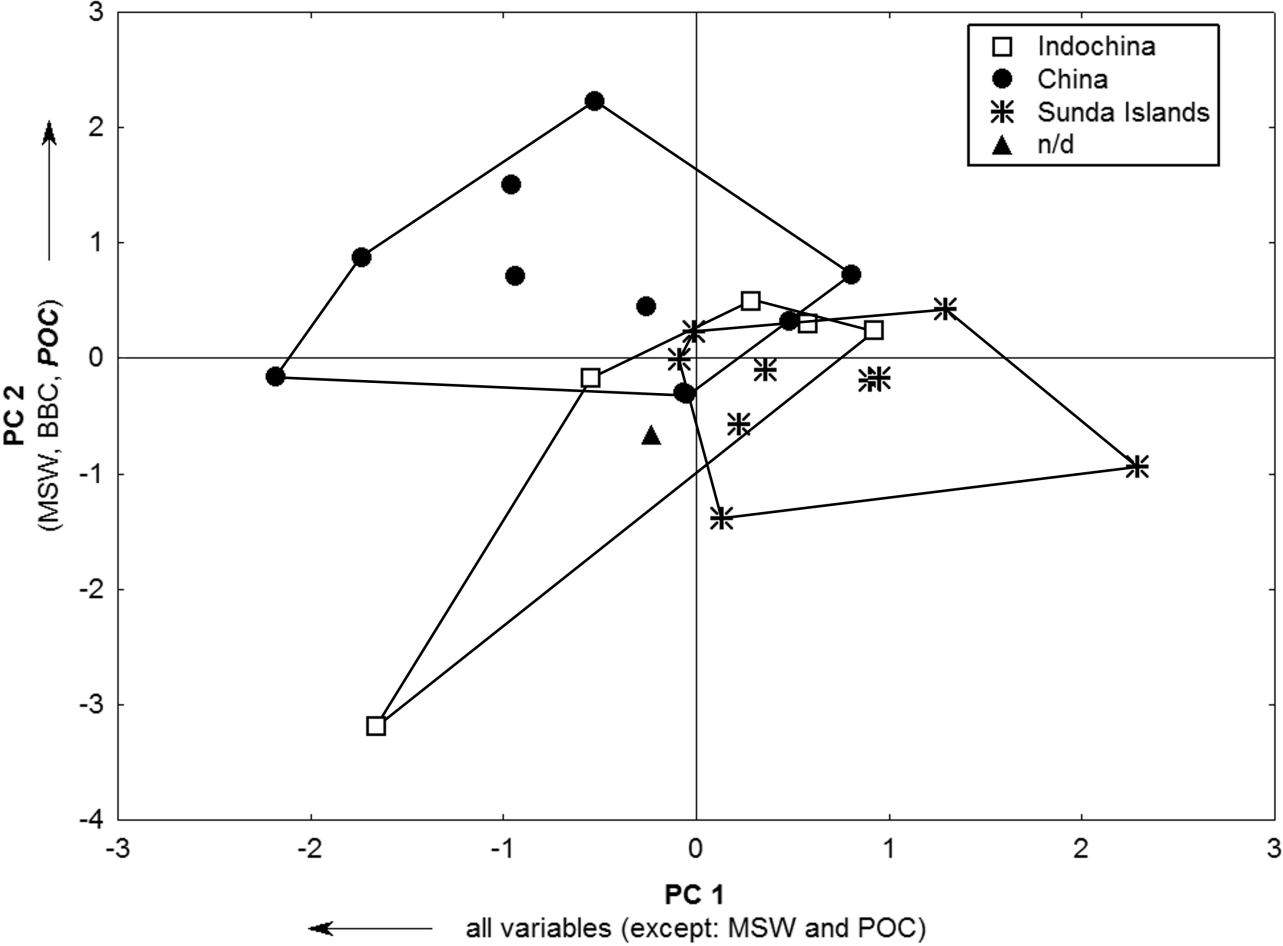
Leopard cat skull PCA morphospace across Asian regions. Bivariate plot of 25 *P. bengalensis* according to individual principal component scores, considering three major geographical regions. Arrows indicate the direction of the contribution of variable loadings to the respective principal components (PCs). Subregions are identified for each individual.

The PCA of the skull measurements included all specimens from the three geographical regions and one with no precedence data (n/d). The first three principal components (PCs) explained 85.6% of the total variation (eigenvalues: PC1 = 14.4; PC2 = 1.4; PC3 = 1.3). All skull measurements but Masseteric scar width (MSW) and Postorbital constriction (POC) are associated with the first PC. According to individual PC scores, there is a size gradient along PC1. Chinese specimens have the largest skulls, contrasting with those from Sunda Islands. PC2 denotes the larger Postorbital constriction (POC), Masseteric scar width (MSW), and Breadth of braincase (BBC) of the Chinese individuals, whereas, the individuals from the Sunda Islands seem to have narrower braincases (BBC and POC). Indochinese specimens are in an intermediary, overlapping position, except for one larger and older individual with a markedly narrow POC. The morphological variation explained by PC3 is quite small (6.3%) and geographical morphotypes overlap (Fig. 1).

**Figure 2:**
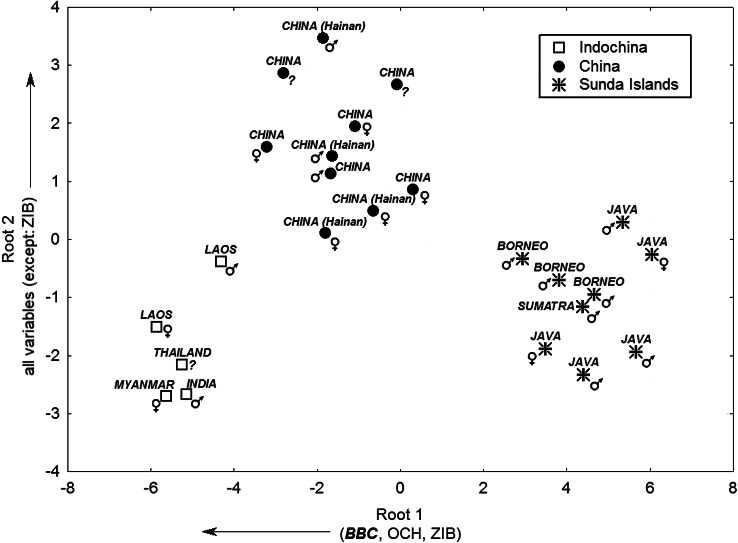
Leopard cat skull DFA morphospace across Asian regions. Bivariate plot of 24 *P. bengalensis* according to individual discriminant function scores, considering three major geographical regions. Arrows indicate the direction of the contribution of variable loadings to the respective canonical roots. Subregions are identified for each individual. Sexes are identified as *males* = ♂, *females* = ♀, and *indeterminate* = ?.

The results of DFA indicate a multivariate difference between the skull measurements of males and females (Wilks’ Lambda: 0.29; *F*_9,12_ = 3.32; *p* < 0.03) considering the whole sample (Fig. 2). Only one canonical root was obtained after forward stepwise selection of the most relevant variables to the discrimination between the sexes (Table 2). Scores of males suggest a larger skull than females. However, the DFA root structure indicates a broader Postorbital constriction (POC) as a particular feature among females. The small sample size hindered any attempt to perform an analysis of sexual dimorphism with regional sub-samples.

**Table 2:** Skull measurements in Discriminant Function Analysis—Sexual dimorphism. Variable loadings of the first canonical root after a forward stepwise DFA of sexual dimorphism based on skull measurements of 22 individuals.

Skull measurement	Canonical root 1
JWM_1_	−**0.25**†
BBC	0.12
ZIB	−**0.16**†
TFL	−0.06
POC	**0.18**†
RWP^2^	−0.01
JL	−**0.16**†
JHM_1_	−**0.23**†
MSW	−0.04

**Notes.**

High variable loading values are highlighted (†).

A negative sign indicates a negative contribution of the variable to the root.

With the three major geographical domains (China, Indochina, and Sunda Islands region) included as an *a priori* classification, a marked discrimination among skull morphological spaces was obtained (Wilks’ Lambda: 0.02, *F*_20,24_ = 8.48, *p* < 0.00001). Two canonical roots accounted for the group discrimination (Table 3) based on the Mahalanobis distance indicating three morphological groups (Table 4). Chinese leopard cats (from both continental China and Hainan Island) are clustered together due to the larger size of their skulls, based on the second canonical root (Fig. 2). Breadth of braincase (BBC), Occipital height (OCH), and Zygomatic arches internal breadth (ZIB) are the main sources of discrimination between groups, according to the first canonical root. Individuals from the Sunda Islands have a narrower and lower-profiled skull while, in contrast, Indochinese forms have skulls with broader faces and braincases and an elevated occipital region. This result is consistent with the PCA analysis, which indicated a narrow BBC among the Sunda Island specimens. The Chinese cluster lies between the other two, denoting an intermediary skull pattern. The Indochina cluster corresponds to the leopard cat subspecies *P. b. bengalensis*. Two small clusters, Borneo and Java, could be recognized in the Sunda Islands, corresponding to the subspecies *P. b. borneoensis* and *P. b. javanensis*, respectively. The smallness of the Sumatran leopard cat skull sample precludes comments on the *P. b. sumatranus* subspecies morphological space. On the other hand, there is no clear difference between the skulls of the individuals from mainland China (*P. b. chinensis*) and those from Hainan Island (*P. b. alleni*).

**Table 3:** Skull measurements in Discriminant Function Analysis—Geographical variation. Variable loadings of the first two canonical roots after a forward stepwise DFA of geographical variation based on skull measurements of 22 individuals.

Skull measurement	Canonical root 1	Canonical root 2
BBC	−**0.31**†	**0.45**†
POC	0.01	**0.24**†
OPL	−0.07	**0.21**†
MMA	−0.07	**0.26**†
OCH	−**0.14**†	**0.20**†
ZIB	−**0.14**†	0.12
JL	0.07	**0.20**†
MSW	0.02	**0.26**†
CBL	−0.11	**0.22**†
MFL	−0.09	**0.25**†

**Notes.**

High variable loading values are highlighted (†).

A negative sign indicates a negative contribution of the variable to the root.

**Table 4:** Post hoc analysis discriminating leopard cat skulls from the three main geographical regions. Squared Mahalanobis Distance *p*-values after a forward stepwise DFA of geographical variation based on skull measurements of 24 individuals according to geographic origin.

	Indochina Peninsula	China	Sunda Islands
Indochina Peninsula		0.01*	0.00001***
China	0.01*		0.0001***
Sunda Islands	0.00001***	0.0001***	

**Notes.**

Marked *p*-values (*) indicate significant differences between groups.

No major patterns of sexual dimorphism were found (Fig. 2) considering the whole sample. Despite the small sample sizes, the same was observed in the three major clusters. This could be interpreted as a smaller influence of sexual dimorphism in the structure of the morphological space of leopard cat skulls, in comparison to the geographical/taxonomical influence.

### Analysis of Allen’s (1938) full data

Nine skull and teeth measurements taken by Allen (1938) were analyzed by univariate non-parametrical statistical methods. Only a minor problem of heteroscedasticity was found in the measurement Median length of nasals (Levene’s test *p* = 0.03), despite the small sample size. The sample assembled by Allen (1938) included one female *P. b. bengalensis* and 20 individuals of *P. b. chinensis* of both sexes. However, seven individuals were later reclassified as *P. b. alleni* (♀*n* = 3, ♂*n* = 4), since they came from the Chinese Island of Hainan. A Mann–Whitney *U* test including all individuals except the single *P. b. bengalensis* (♀*n* = 11, ♂*n* = 9), indicated skull sexual dimorphism in the following measurements: Greatest length (*p* < 0.04), Basal length (*p* < 0.02), Zygomatic width (*p* < 0.01), Mastoid width (*p* < 0.05), Median length of nasals (*p* < 0.02), and Lower cheek teeth (*p* < 0.01).

Despite Allen’s (1938) small sample, *P. b. chinensis* (♀*n* = 8, ♂*n* = 5) showed sexual dimorphism in all nine skull and teeth measurements (*U*_2,13_ = 0.0–6.0, *p*-values ranging from <0.01 to <0.05). Sexual differences found in skull morphology in Allen’s (1938) sample corroborate some findings obtained from our own sample (considering some similar measurements): sexual dimorphism in Mastoid breadth (MB) and Zygomatic arches internal breadth (ZIB) are equivalent to Allen’s Mastoid width and Zygomatic width. Unfortunately, the small sample of *P. b. alleni* does not allow for comparisons.

Multivariate analysis (DFA) considering all individuals of Allen’s sample (except *P. b. bengalensis*) indicated sexual size dimorphism: male skulls were slightly larger than female (Wilks’ Lambda: 0.23; *F*_9,10_ = 3.81; *p* < 0.03). Using the subspecies as an a priori classification, the DFA indicated a difference (Wilks’ Lambda: 0.34; *F*_8,13_ = 3.21; *p* < 0.03) between *P. b. chinensis* (*n* = 15) and *P. b. alleni* (*n* = 7) based on eight of Allen’s (1938) skull measurements (Lower cheek teeth was excluded since it was not available for all specimens). Seven of these variables (excluding Upper cheek teeth) showed high loadings associated with the single canonical root. Based on the scores of each group, *P. b. chinensis* had a larger overall skull size than *P. b. alleni.* This result from Allen’s data improves discrimination between the two subspecies, which was not clearly observed in the Chinese cluster obtained with our original data.

### Analysis of Pocock’s (1939) full data

The sample assembled by Pocock (1939), considering subspecies and sexes, was not large enough to support indubitable conclusions (♀*n* = 4, ♂*n* = 9), and therefore the results of the statistical analyses should be interpreted as trends. Sexual dimorphism was tested by combining the samples from Northern India (Kumaun) and Nepal (*P. b. horsfieldii*) and that from Southern peninsular India (*P. b. bengalensis*). The single specimen of *P. b. trevelyani* from the slopes of Murree (Northern Pakistan) was excluded from the analyses. A Mann–Whitney *U* test based on five skull measurements and the length of the fourth upper premolar (Maxillary width and Mandibular length were excluded because they were missing for some specimens) indicated sexual skull dimorphism in Total length [of skull] (*U*_2,13_ = 4.0, *p* < 0.03), Condyle-basal length (*U*_2,13_ = 4.0, *p* < 0.03), and Zygomatic width (*U*_2,13_ = 2.5, *p* < 0.01), males presenting higher mean values on these measurements than females.

The northern form *P. b. horsfieldii* (*n* = 8) only had a larger PM^4^ than the southern form *P. b. bengalensis* (*n* = 5) in the Mann–Whitney test (*U*_2,13_ = 5.5; *p* < 0.03). This analysis suggests skull morphology is less important than external body features, such as coat length and thickness, and color pattern, for the diagnosis of these subspecies. Small sample size hindered reliable DFA results for both sexual dimorphism and subspecific comparisons.

Pocock’s (1939) external body measurements of leopard cats (Head-body length, Tail length, and Hind foot length) were also compared with a Mann–Whitney *U* test. The small sample allowed for simple comparisons between the sexes only for a combined sample of *P. b. bengalensis* and *P. b. horsfieldii*. Significant differences were observed only for the hind foot; males were on average 12.7 mm larger than females (*U*_2,9_ = 0.0; *p* < 0.02). The other measurements showed no significant sex-related differences (Mann–Whitney *p*-values ranging from >0.40 to >0.90). Despite the small sample, the high *p*-values obtained in the comparison between *P. b. horsfieldii* and *P. b. bengalensis* suggest the overall similarity between the external body measurements of these subspecies.

### Analysis of Heptner & Sludskii’s (1992) summary data

The *t*-test for summary data comparing adult males (*n* = 12) and females (*n* = 6) of *P. b. euptilurus*, using six skull measurements, indicated marked sexual dimorphism (Table 5). The same was observed among subadult forms (♀*n* = 9–12, ♂*n* = 7–8, according to the measurements available, Table 6). Subadult and adult males had larger skull measurements than females, with the exception of Postorbital width, for which there was no difference between the sexes (*p* > 0.26). Thus, this feature has an allometric relationship to other skull dimensions in males and females in both age classes. The anterior part of the braincase (defined by Postorbital width) of these Amur leopard cat females appears to be broader than in the males. Ontogenetic comparisons between subadult and adult Amur leopard cats showed that adult males were larger than subadult males in all skull measurements with the exception of Postorbital width, which was broader in the subadults (*p* < 0.001). Ontogenetic differences among females also indicate larger skull dimensions in adults, except for Interorbital width (*p* > 0.12) and Postorbital width (*p* < 0.0001), which are broader in subadults. Thus, Postorbital width seems to be hypertrophied in subadult forms. This feature remains more developed among adult females than among adult males. This is similar to the result found with our original data on males and females from several southeastern Asian regions, in which females were broader across the Postorbital constriction (POC) than males.

**Table 5:** Skull sexual dimorphism in adult Amur leopard cats. Heptner & Sludskii (1992) data with *p*-values after a *t*-test for summary data for adult Amur leopard cats (*P. b. euptilurus*).

Skull measurement (mm)	♂*n*	♂ Mean	♂ SD	♀*n*	♀ Mean	♀ SD	*p*
Greatest length	12	108.70	2.72	6	99.00	0.94	0.00001***
Condylobasal length	12	100.10	2.00	6	91.30	0.58	0.00001***
Zygomatic breadth	11	71.70	1.44	5	62.40	1.44	0.00001***
Interorbital width	12	17.50	0.64	6	15.40	0.66	0.00001***
Postorbital width	11	25.50	1.04	6	26.00	0.38	0.28
Length of upper tooth row with C^1^	12	32.70	0.68	6	30.40	0.30	0.00001***

**Notes.**

Marked *p*-values indicate a significant difference between the sexes (*).

**Table 6:** Skull sexual dimorphism among subadult Amur leopard cats. Heptner & Sludskii’s (1992) data with *p*-values after a *t*-test for summary data for subadult Amur leopard cats (*P. b. euptilurus*).

Skull measurement (mm)	♂*n*	♂ Mean	♂ SD	♀*n*	♀ Mean	♀ SD	*p*
Greatest length	7	95.60	1.80	9	91.00	2.56	0.001**
Condylobasal length	7	88.90	1.56	10	84.20	1.98	0.0001***
Zygomatic breadth	8	60.40	1.46	9	58.00	1.68	0.01*
Interorbital width	8	14.90	0.46	11	14.00	0.68	0.01*
Postorbital width	8	28.00	0.96	11	27.60	0.54	0.26
Length of upper tooth row with C^1^	8	30.50	0.52	12	28.40	0.56	0.00001***

**Notes.**

Marked *p*-values indicate a significant difference between the sexes (*).

### Analysis of Groves’ (1997) summary data

Despite the relatively large sample assembled by Groves (1997), his data were subdivided among seven locations and subspecies. They allowed for exploratory comparisons of skull measurements between males and females from Mainland [Indochina] (*P. b. bengalensis*), Sumatra (*P. b. sumatranus*), Java (*P. b. javanensis*), and Borneo (*P. b. borneoensis*). The results of the sexual dimorphism analysis of these subspecies are presented on Table 7. Despite the small samples for each sex in these locations, an unexpected pattern could be observed in the variation in skull morphology between the sexes: in *P. b. bengalensis* from Mainland, sexual dimorphism was present in Greatest skull length and Bizygomatic breadth; no sexual dimorphism was observed in the three skull features in *P. b. sumatranus* or in *P. b. borneoensis*; and marked sexual dimorphism was observed in the three skull measurements (Greatest skull length, Condylobasal length, Bizygomatic breadth) among *P. b. javanensis* individuals. Therefore, in addition to the morphological differences cited by Groves (1997) for these subspecies in Condylobasal length ratios, our analyses indicate different skull shape patterns among them due to sexual dimorphism. These variations range from well-marked differences, such as the larger size of male skulls, to no differences in skull morphology between the sexes at all. These results should therefore be regarded as trends, whereas the evaluation of full data could be more informative.

**Table 7:** Sexual dimorphism in leopard cat skulls by geographical regions. Groves’ (1997) data with *p*-values after a *t*-test for summary data for adult leopard cats according to location: Mainland [Indochina] (*P. b. bengalensis*), Bali and Java (*P. b. javanensis*), Borneo (*P. b. borneoensis*), Sumatra (*P. b. sumatranus*), Negros (*P. b. rabori*), and Palawan (*P. b. heaneyi*).

Location	Skull measurement (mm)	♂*n*	♂ Mean	♂ SD	♀*n*	♀ Mean	♀ SD	*p*
Mainland								
	Greatest skull length	3	95.00	3.00	6	89.70	2.73	0.03*
	Condylobasal length	3	84.00	2.65	6	81.80	1.60	0.16
	Bizygomatic breadth	3	63.00	2.08	6	60.20	0.75	0.02*
Sumatra								
	Greatest skull length	7	89.90	3.08	4	87.00	2.16	0.13
	Condylobasal length	7	80.40	3.55	4	78.30	2.63	0.33
	Bizygomatic breadth	7	59.70	3.05	4	56.90	2.72	0.16
Java								
	Greatest skull length	12	87.50	2.95	9	82.90	3.55	0.01*
	Condylobasal length	12	80.80	3.77	9	72.80	4.15	0.001**
	Bizygomatic breadth	12	56.20	2.78	9	51.90	2.88	0.01*
Borneo								
	Greatest skull length	8	86.80	2.45	7	85.10	2.97	0.25
	Condylobasal length	8	79.40	2.20	6	77.10	2.69	0.10
	Bizygomatic breadth	8	58.30	1.44	8	56.60	2.97	0.17

**Notes.**

Marked *p*-values indicate a significant difference between the sexes (*).

Inter-subspecific analyses were conducted for all groups with *n* ≥ 3. Males and females were compared in separate analyses for each skull measurement proposed by Groves (1997). Male samples included individuals from Negros (*n* = 3), Palawan (*n* = 5), [Indochina] Mainland (*n* = 3), Sumatra (*n* = 7), Java (*n* = 8), Borneo (*n* = 12), and Bali (*n* = 4). Female analyses included samples from Mainland (*n* = 6), Sumatra (*n* = 4), Java (*n* = 9), and Borneo (*n* = 7). These analyses were intended to bring a more formal methodological approach to the data and to provide insights into subspecific differences among leopard cats.

The ANOVA based on summary data for Greatest skull length found a difference among males (*F*_6, 35_ = 7.32; *p* < 0.0001); Bonferroni’s post-hoc test results are presented in Table 8. The Greatest skull length of *P. b. bengalensis* was significantly larger than most of other subspecies compared. The Sumatran subspecies (*P. b. sumatranus*) also had a larger Greatest skull length than Balinese individuals of *P. b. javanensis*. Females showed differences between groups (*F*_3, 22_ = 6.29; *p* < 0.01), but Bonferroni’s post-hoc test only detected the significantly larger size of *P. b. bengalensis* from [Indochina] Mainland in relation to *P. b. javanensis* from Java (*p* < 0.01).

**Table 8:** Greatest skull length variation in male leopard cats by geographical regions. Bonferroni’s post hoc test between male populations of Southeastern Asian leopard cats after an ANOVA on Groves’ (1997) summary data for Greatest skull length. Locations and subspecies are: Mainland [Indochina] (*P. b. bengalensis*), Bali and Java (*P. b. javanensis*), Borneo (*P. b. borneoensis*), Sumatra (*P. b. sumatranus*), Negros (*P. b. rabori*), and Palawan (*P. b. heaneyi*).

	Bali	Borneo	Java	Mainland	Negros	Palawan
Borneo	1.00	–	–	–	–	–
Java	0.26	1.00	–	–	–	–
Mainland	0.0001***	0.001**	0.01*	–	–	–
Negros	0.42	1.00	1.00	0.09	–	–
Palawan	1.00	1.00	1.00	0.001**	1.00	–
Sumatra	0.01*	0.61	1.00	0.17	1.00	0.09

**Notes.**

Marked *p*-values indicate significant differences between location morphotypes (*).

Condylobasal length presented no difference among males (*F*_5, 33_ = 2.23; *p* > 0.07). However, females showed significant differences between groups (*F*_3, 21_ = 28.91; *p* < 0.00001; see Table 9). Significant differences were detected between *P. b. bengalensis* and *P. b. borneoensis*, between *P. b. bengalensis* and *P. b. javanensis* from Java, and between *P. b. bengalensis* and *P. b. sumatranus*, indicating a larger Condylobasal length in females from Mainland in relation to those from the southeastern Asian islands. The post-hoc test also suggested a possible difference in Condylobasal length between the smaller *P. b. javanensis* and larger *P. b. sumatranus* (*p* = 0.05).

**Table 9:** Condylobasal length variation in female leopard cats by geographical regions. Bonferroni’s post hoc test between female populations of Southeastern Asian leopard cats after an ANOVA on Groves’ (1997) summary data for Condylobasal length. Locations and subspecies are: Mainland [Indochina] (*P. b. bengalensis*), Borneo (*P. b. borneoensis*), Sumatra (*P. b. sumatranus*).

	Borneo	Java	Mainland
Java	0.10	–	–
Mainland	0.0001***	0.0000001***	–
Sumatra	1.00	0.05*	0.001**

**Notes.**

Marked *p*-values indicate significant differences between location morphotypes (*).

Summary data ANOVA results for Bizygomatic breadth indicated differences among males (*F*_6, 35_ = 8.80; *p* < 0.000001; see Table 10). The analysis showed that continental *P. b. bengalensis* had larger skulls than *P. b. javanensis* (Java and Bali) and *P. b. heaneyi* (Palawan). Individuals of *P. b. sumatranus* seemed to have broader skulls than *P. b. javanensis* from Bali (but not from Java) and *P. b. heaneyi* from Palawan. Bornean leopard cats (*P. b. borneoensis*) had broader skulls than Balinese *P. b. javanensis*. Bizygomatic breadth differed among females as well (*F*_3, 23_ = 13.16; *p* < 0.001). This feature was significantly different between females of *P. b. javanensis* and *P. b. borneoensis* (*p* < 0.01), and between *P. b. javanensis* and *P. b. sumatranus* (*p* < 0.02), and markedly different between *P. b. bengalensis* and *P. b. javanensis* (*p* < 0.0001).

**Table 10:** Bizygomatic breadth variation in male leopard cats by geographical regions. Bonferroni’s post hoc test between male populations of Southeastern Asian leopard cats after an ANOVA on Groves’ (1997) summary data for Bizygomatic breadth. Locations and subspecies are: Mainland [Indochina] (*P. b. bengalensis*), Bali and Java (*P. b. javanensis*), Borneo (*P. b. borneoensis*), Sumatra (*P. b. sumatranus*), Negros (*P. b. rabori*), and Palawan (*P. b. heaneyi*).

	Bali	Borneo	Java	Mainland	Negros	Palawan
Borneo	0.01*	–	–	–	–	–
Java	0.15	1.00	–	–	–	–
Mainland	0.0001***	0.07	0.001**	–	–	–
Negros	0.16	1.00	1.00	0.11	–	–
Palawan	1.00	0.22	1.00	0.001**	1.00	–
Sumatra	0.001**	1.00	0.13	0.60	1.00	0.02*

**Notes.**

Marked *p*-values indicate significant differences between location morphotypes (*).

### Analysis of Lim’s (1999) full data

In his work on the distribution, food habits and parasites of *P. b. bengalensis* in Peninsular Malaysia, Lim (1999) included external body measurements (Head & Body, Tail, and Hind Foot) of 12 males and eight females. The data had no problems of heteroscedasticity and, despite the small sample, no major problems of frequency distribution. On average, the hind feet of males were 14.2 mm longer than those of females (*U*_2,20_ = 6.0; *p* < 0.001), showing marked sexual dimorphism. This result was similar to that found with Pocock’s (1939) data, though that sample was a combined set of *P. b. bengalensis* and *P. b. horsfieldii*. Other external body measurements did not show significant differences (*p*-values > 0.25), denoting little sexual dimorphism in the external dimensions of *P. b. bengalensis.* Weight was not included in the analysis due to the susceptibility of this trait to seasonal fluctuations. Sexual dimorphism was evidenced when assessed through DFA (Wilks’ Lambda: 0.35; *F*_3,16_ = 9.92; *p* < 0.001); Hind Foot, however, had a markedly higher loading in the structure of the canonical root (−0.84) than the other two variables (≈ − 0.17), denoting the importance of this variable in the discriminant function. The larger skull of male *P. b. bengalensis* observed in the analysis of Groves’ (1997) summary data seemed to have only a minor influence on the external Head & Body measurements.

### Analysis of Rajaratnam’s (2000) full data

The number of Sabah leopard cats (*P. b. borneoensis*) with external body measurements presented by this author was quite limited (♀*n* = 4, ♂*n* = 3). Head-and-Body and Tail lengths, when submitted to an exploratory Mann–Whitney *U* test, showed no significant differences between sexes. In spite of the small sample size, the possible absence of sexual dimorphism seems to be in accordance with the analysis of Groves’ (1997) summary data, which also indicated an absence of sexual variation in skull measurements in *P. b. borneoensis*.

### Analysis of full data from Grassman et al. (2005)

The paper by Grassman et al. (2005) on spatial organization and diet included a comprehensive external morphometric description of *P. b. bengalensis* from Thailand (Weight, Head and Body, Tail, Hind Foot, Ear, and Upper Canine). However, the sample sizes of adult males and females were quite asymmetrical (♀*n* = 4, ♂*n* = 13). We excluded Weight from our analysis due to the intrinsic variation of this parameter with season, and Upper Canine due to the absence of values in some individuals. No significant differences between males and females were found in Head and Body (*p* > 0.16), Tail (*p* > 0.10), Hind Foot (*p* > 0.08), or Ear (*p* > 0.70). The same was noted in the DFA (Wilks’ Lambda: 0.55; *F*_4,12_ = 2.43 *p* > 0.11).

### Analysis of combined full data from Pocock (1939), Lim (1999) and Grassman et al. (2005)

These authors presented full tables of external body measurement values of *P. b. bengalensis* from India and Myanmar, Peninsular Malaysia, and Thailand, respectively. Their data was reorganized in a single table of Head and Body length, Tail length and Hind Foot length. The combined sample of 29 male and 14 female individuals was homoscedastic for all three variables, although Hind Foot length had a non-normal distribution (Shapiro–Wilk’s *W* test, *p* < 0.02). Sexual dimorphism was absent in Head and Body length and Tail length (Mann–Whitney *U* test; *p* > 0.08, for both variables), but the markedly larger size of males’ Hind Foot length (*U*_2,43_ = 35.5; *p* < 0.0001) was confirmed.

As a validation test, a comparison between the authors’ data was made in order to verify if the external body measurements had been taken in a standard way. There were no significant differences in the way the authors had taken Head and Body and Hind Foot lengths (Kruskal–Wallis ANOVA, *p*-values >0.40 and >0.17, respectively). However, there was a marked difference in Tail length measured by the authors (*H*_3,43_ = 24.1; *p* < 0.00001). Dunn’s post-hoc test found that Lim’s Tail length measurement was significantly different from Pocock’s (*p* < 0.0001) and from Grassman et al.’s (*p* < 0.001), albeit no difference was found between Pocock’s and Grassman et al.’s measurements (*p* > 0.35). Therefore, Tail length was reanalyzed excluding Lim’s (1999) measurements to evaluate the occurrence of sexual dimorphism. Once again, we did not find any significant difference (Mann–Whitney *U* test) between male (*n* = 17) and female (*n* = 6) tail lengths in *P. b. bengalensis*. The differences found in Tail length between the authors could mean some particularity in the way Lim took the tail measurement, or some morphotypical variation in the specimens from Peninsular Malaysia. In the latter hypothesis, Peninsular Malaysian *P. b. bengalensis* would have shorter tails than those from the more continental areas in Indochina.

Head and Body length and Tail length of *P. b. bengalensis* (samples of Pocock, 1939; Grassman et al., 2005) and *P. b. borneoensis* (sample of (Rajaratnam, 2000)) were compared to evaluate subspecific variations in these two traits. A combined sample of male and female *P. b. bengalensis* (*n* = 23) and *P. b. borneoensis* (*n* = 7) was compared. Marked size variation was observed between the two subspecies in both Head and Body length (*U*_2,30_ = 1.5; *p* < 0.00001) and Tail length (*U*_2,30_ = 6.0; *p* < 0.0001). *P. b. bengalensis* was about 19% larger in Head and Body length and about 23% larger in Tail length than *P. b. borneoensis*. One could conjecture that the way Rajaratnam (2000) took his measurements might have caused this variation as well. However, these findings are in accordance with our analysis of Groves’ (1997) summary data, which showed that Greatest Skull length was significantly larger in *P. b. bengalensis* from Indochina than in *P. b. borneoensis*.

### Analysis of summary data presented by Sunquist & Sunquist (2002)

We consulted the originals of most of the studies cited by Sunquist & Sunquist (2002). The authors presented only summary information on external body measurements, sometimes based on one or two individuals. Therefore, a new table was organized including values listed by Sunquist & Sunquist (2002), but also including new values calculated from other works by the same authors. Standard deviations, where not given, were calculated based on the Empirical Rule (Sternstein, 2005; Utts & Heckard, 2006). Since Tail length might be a source of bias depending on the authors, the meta-analysis of several leopard cat subspecies was based only on male Head-and-Body length (Table 11).

**Table 11:** Body size variation in male leopard cats by geographical region. Head-and-Body summary statistics for leopard cat subspecies, based on published studies included in Sunquist & Sunquist’s (2002) table.

Subspecies	Location	Reference	Mean (mm)	Min (mm)	Max (mm)	SD	♂*n*
*P. b. chinensis*	China	Shaw/Shou (1962)†	584	540	660	≈24	4
*P. b. chinensis*	Eastern China	Tan (1984)	488.7	450.0	560.0	≈22	11
*P. b. trevelyani*	Pakistan	Pocock (1939)*	548.6	–	–	–	1
*P. b. horsfieldii*	Northern India, Nepal	Pocock (1939)*	546.1	538.5	553.7	–	2
*P. b. bengalensis*	India, Myanmar	Pocock (1939)	547.4	508.0	614.7	46.6	4
*P. b. bengalensis*	Thailand	Grassman (1998)†*	583	570	600	–	3
*P. b. bengalensis*	Thailand	Grassman et al. (2005)	573.5	500.0	640.0	42.0	13
*P. b. borneoensis*	Malaysian Borneo	Rajaratnam (2000)	473.8	455	500	18.9	4
*P. b. bengalensis*	Peninsular Malaysia	Lim (1999)	545.8	430	625	66.4	12
*P. b. euptilurus*	Amur	Heptner & Sludskii (1992)	655	600	750	≈30	9

**Notes.**

Marked references (*) were excluded from the analysis due to small sample size.

Values of SD were recalculated based on full data from the original papers or calculated by means of the empirical rule (≈).

Data from references marked (†) were not checked against the original papers and were based solely on the figures presented by Sunquist & Sunquist (2002).

An ANOVA on summary data using locations as grouping variables indicated a significant difference between groups (*F*_6,50_ = 15.9; *p* < 0.00001). Bonferroni’s post-hoc test results are presented in Table 12. *Prionailurus b. euptilurus* from Amur showed a larger Head and Body length than almost all other subspecies, except for *P. b. chinensis* cited by Sunquist & Sunquist (2002), based on Shaw/Shou’s work (*apud* Sunquist & Sunquist, 2002), with location specified only as “China.” This sample may well have included very different forms, since they had a greater mean value than the larger sample from Eastern China cited by Tan (1984). The *P. b. chinensis* from “China” (based on Shaw/Shou, 1962, *apud* Sunquist & Sunquist, 2002) had a Head-and-Body length larger than Malaysian-Bornean *P. b. borneoensis*. However Eastern China *P. b. chinensis* seemed to be smaller than the Thai *P. b. bengalensis*. Finally, *P. b. bengalensis* from Thailand had a Head-and-Body length larger than *P. b. borneoensis*. The sample of *P. b. bengalensis* from Lim’s (1999) work did not differ from Rajaratnam’s (2000) *P. b. borneoensis*. This was also observed in a comparison of their original full data (*U*_2,27_ = 38.0; *p* > 0.08).

**Table 12:** Post hoc comparisons of leopard cat body size by geographical region. *P*-values from Bonferroni’s post-hoc test after an ANOVA on summary data for Head-and-Body length. According to the locations mentioned in the papers used for this analysis, individuals from China and Eastern China are *P. b. chinensis*; India/Myanmar, Thailand, and Peninsular Malaysia *P. b. bengalensis*; Malaysian *Borneo P. b. borneoensis*; and Amur *P. b. euptilurus*.

	Amur	China	Eastern China	India and Myanmar	Malaysian Borneo	Peninsular Malaysia
China	0.17	–	–	–	–	–
Eastern China	0.000001***	0.01*	–	–	–	–
India/Myanmar	0.01*	1.00	0.47	–	–	–
Malaysian Borneo	0.000001***	0.01*	1.00	0.38	–	–
Peninsular Malaysia	0.00001***	1.00	0.05	1.00	0.11	–
Thailand	0.001**	1.00	0.001**	1.00	0.01*	1.00

**Notes.**

Marked *p*-values indicate significant differences between location morphotypes (*).

## Discussion

Several issues have contributed to the lack of precise information about sexual dimorphism in leopard cats. The same could be said in relation to the morphometry of geographical morphotypes across their areas of occurrence. First of all, historically, leopard cat sample sizes have been quite small. Even objective attempts to assemble more representative material faced problems with the origin of the individuals. For instance, the comprehensive sample of 72 individuals assembled by Groves (1997) to perform skull morphometric analysis is split into seven sub-groups with *n*-values ranging from one to, at most, 12 individuals according to the location and sex. Although the leopard cat is quite a common cat in southern Asia (Guggisberg, 1975), wild cats as a whole are elusive in nature, and skeletons and skins are scarce in collections. The second difficulty regarding descriptions of leopard cats’ variability is their wide distribution in central and southeastern Asia, including the Malaysian, Indonesian and Philippine islands. Moreover, as shown here, regional morphotypes have different degrees of sexual dimorphism. Therefore, despite isolated efforts over the last 86 years (e.g., Allen, 1929) to collect specimens, assemble material and describe regional subspecies, the information about sex, age, and geographical variation is still scattered and heterogeneous. Finally, there is not a unified morphometric methodology to ensure a sound comparative discussion on the same morphological standards.

### Sexual dimorphism among leopard cat subspecies

Although there is subtle skull and body sexual dimorphism among leopard cats, it does not occur in all subspecies. As a general pattern for the species based on our original data, male *P. bengalensis* skulls are larger than females’, especially in the robustness of lower jaw (Jaw width and Jaw height at the first lower Molar—JWM_1_ and JHM_1_—and Jaw length—JL), and width of the skull (Zygomatic breadth—ZIB). However, leopard cat females show a larger width across the Postorbital constriction (POC) than males according to the first canonical root. Statistical analysis of leopard cats from Allen’s (1938) data revealed the greater size of male skull measurements among Continental China specimens (*P. b. chinensis*). Nevertheless, leopard cats from the Hainan Island (*P. b. alleni*) seem to have much more subtle or even no sexual dimorphism in their skull features. This suggests two patterns of skull sexual dimorphism.

On the other hand, leopard cats from Nepal, Myanmar, and Northern and Southern India (*P. b. horsfieldii* and *P. b. bengalensis*, respectively) also showed the same “males-larger-than-females” skull pattern, based on analysis of Pocock’s (1939) full data. *Prionailurus b. euptilurus* from the Amur region also showed the same sexual dimorphism in skull size in the analysis of Heptner & Sludskii’s (1992) data. However, no difference was detected in Postorbital width between the sexes, denoting the allometric relation of this feature to overall skull size in males and females. The well-developed postorbital region found in these Amur leopard cat females corroborates our findings for Postorbital constriction (POC) and highlights the relevance of this feature as a sexual dimorphic feature of leopard cat skulls. This pattern seems to be ontogenetic, in view of the well-developed Postorbital width among subadult individuals. It is likely, therefore, that two separate ontogenetic pathways exist during sexual maturation in male and female leopard cats regarding the postorbital region. An allometric relationship between felid braincase dimensions and body size is also discussed by Meachen-Samuels & Van Valkenburgh (2009) in relation to different metabolic demands. One could suppose that the large POC in female skulls follows this pattern. However, according to our PCA results, larger geographical morphotypes (Chinese forms, for instance) also have a broad POC, since this measurement has a high loading in the composition of PC2 (Fig. 1). Therefore, some other morphofunctional or physiological mechanism may be influencing this metric relationship in leopard cats.

Groves (1997) pointed out differences in the ratio of Condylobasal length between males and females related to the geographical origin of leopard cat populations in Peninsular Malaysia and the Indonesian and Philippine islands. Our analysis of Groves’ (1997) summary data added a new perspective to his findings, indicating a greater skull size of male *P. b. bengalensis* and *P. b. javanensis*, but also the absence of significant skull sexual dimorphism in *P. b. sumatranus* and *P. b. borneoensis* populations.

In spite of the sexual variation in skull morphology presented by some subspecies, traditional external body measurements point to a much more subtle difference between male and female leopard cats. In the relatively well-sampled data from Lim’s (1999) study, only Hind Foot length showed sexual variation in peninsular Malaysian *P. b. bengalensis*. This pattern is consistent with the analysis of pooled data from Pocock (1939) and Grassman et al. (2005) for populations of the same subspecies from India/Myanmar and Thailand, respectively. In both analyses, the hind foot of males was 9.3 mm–14.2 mm larger than that of females. Similar hind foot size variation (12.7 mm) was also found in Pocock’s (1939) sample, including *P. b. bengalensis* and *P. b. trevelyani* individuals. The analysis of only Grassman et al.’s (2005) sample (adults: ♀*n* = 4, ♂*n* = 13), however, did not show any sexual dimorphism in *P. b. bengalensis*.

The analysis of Groves’ (1997) summary data did not show skull sexual dimorphism in *P. b. borneoensis*. The same pattern was present in external body measurements according to the exploratory analysis of Rajaratnam’s (2000) data. Unfortunately, Rajaratnam’s (2000) external body measurements were composed only of Head and Body length, Tail length and Weight (not analyzed), and did not included hind foot measurements to further comparisons.

Despite some differences in skull structure and external body measurements in certain subspecies, sexual dimorphism in leopard cats does not seem to be a remarkable aspect of their biology. This pattern is quite unusual among felids, since sexual dimorphism in size and shape is noteworthy throughout their evolutionary lineage (Meachen-Samuels & Van Valkenburgh, 2009; Sicuro & Oliveira, 2011; Christiansen & Harris, 2012; Farhadinia et al., 2014). This is found in external as well as cranial and post-cranial skeletal features in several felid species. Marked sexual dimorphism implies different social and even ecological roles between the sexes (Ruckstuhl & Clutton-Brock, 2005). Different social roles can be easily perceived in several home range patterns exhibited by males and females among the Felidae (Sunquist & Sunquist, 2002). Thus, the absence of well-marked sexual dimorphism in leopard cats may have ecological implications. Some ecological data, highlighted below, may support this inference.

According to Rajaratnam’s (2000) data based on five males (adult *n* = 4) and five females (adult *n* = 3), *P. b. borneoensis* males have larger home ranges than females. Rajaratnam et al. (2007) presented new numerical values for male and female Bornean leopard cats’ home ranges, but their sample size was insufficient for statistical comparisons. Mohamed et al. (2013) conjectured about the larger home range of *P. b. borneoensis* males, largely based on the figures provided by Rajaratnam et al. (2007) but, once again, their own sample size was too small for an unequivocal definition of home range size differences between the sexes. Therefore, as occurred with earlier morphometric descriptions of sexual dimorphism in leopard cats, small samples resulted in less than desirable quantifications of their real ecological structure. Nevertheless, Grassman et al. (2005) with relatively well-sampled data (♀ adult *n* = 4, ♀ subadult *n* = 4 and ♂ adult *n* = 10, ♂ subadult *n* = 4) determined that home ranges of both male and female leopard cats (*P. b. bengalensis*) were quite similar in Thailand forests, and they had a social organization marked by loose territoriality. This seems an important fact to compare with the absence of marked sexual dimorphism in this species.

Another remarkable aspect of leopard cat biology came from Frese (1980) and Sunquist & Sunquist (2002). These authors, quoting several anecdotal records collected in zoos, mentioned the notable participation by male leopard cats in the rearing of the young, alongside the females. Nevertheless, there is no information about this parental care behavior in the wild, and such behavior is not usual in other wild felids in zoos. Based on the ecological evidence and zoo records, one could speculate that some evolutionary correlation may exist between the low degree of morphological sexual dimorphism and the behavior displayed by leopard cats of both sexes. Therefore, the low level of sexual dimorphism observed here could provide some insight into the little or no difference between male and female *P. bengalensis* home ranges, and the unusual parental care behavior shown by male leopard cats in specific conditions.

### Geographical and subspecific variations among leopard cats

Our exploratory analysis of original data suggests that skulls of Chinese leopard cats (*P. b. chinensis* and *P. b. alleni*) are larger than those of leopard cat morphotypes from the Sunda Islands (*P. b. borneoensis*, *P. b. javanensis*, and *P. b. sumatranus*) and Indochina (*P. b. bengalensis*). Nevertheless, beyond the size variation, there is a gradient in non-sagittal skull measurements (Breadth of braincase/Zygomatic arches internal breadth, and Occipital height) from the more narrow-headed leopard cats from the Sunda Islands to the more broad-headed leopard cats from Indochina. This analysis also suggested the discrimination of two skull morphological spaces among Sunda Island leopard cats: *P. b. borneoensis* and *P. b. javanensis*. However, it failed to discriminate continental Chinese *P. b. chinensis* from Hainan Island *P. b. alleni*. On the other hand, multivariate analysis of Allen’s (1938) data did allow for the discrimination of *P. b. chinensis* and *P. b. alleni* in skull features. The Continental China sample of *P. b. chinensis* had larger skulls than *P. b. alleni* from Hainan Island. This analysis improves on Allen’s (1938) descriptions, since the author indicated that differences between individuals from different parts of China were too small ((Allen, 1938): page 463).

There are differences in general body appearance between *P. b. bengalensis* and *P. b. horsfieldii*, such as color pattern and fur coat volume. However, the skull morphology based on Pocock’s (1939) data did not show much variation between them, except a longer PM^4^ in *P. b. bengalensis*. Therefore, even taking into account the small basis for comparison, skull features are not the best tool to support the taxonomic diagnosis of these two subspecies.

Analysis of Groves’ (1997) summary data allowed for further insights into skull variation among Malay, Indonesian and Philippine subspecies. The main conclusion is the larger size of Mainland [Indochina] *P. b. bengalensis* skulls compared with most of the island morphotypes. Unfortunately, two of the three measurements taken by the author represent the skull sagittal plane length (Greatest skull length and Condylobasal length), which hinders a thorough approach to the skull shape. Bizygomatic breadth is the only measurement orthogonal to the others. Thus, inferences about skull morphology are restricted to these two main axes (sagittal and transverse). However, some differences found in one measurement but not in another allow for some insight into skull structure variation between the subspecies. Sample sizes from Negros and Palawan are too small for robust statistical morphometrics, let alone for analyses of summary data. Notwithstanding, we found significant differences in the sagittal and transverse planes of the skull between the Palawan leopard cat morphotype (*P. b. heaneyi*) and the Mainland [Indochina] and Sumatran subspecies (*P. b. bengalensis* and *P. b. sumatranus*, respectively), denoting the smaller size of the Palawan skull. This new analysis of Groves’ (1997) results indicated that males showed greater differences in Greatest skull length than in Condylobasal length, suggesting shape variations among the subspecies. Yet, no Bizygomatic breadth variations were found between subspecies, indicating another aspect of skull allometry among the subspecies.

External body measurement comparisons also revealed some clues about subspecific differences. A comparison of Head-and-Body length and Tail length between *P. b. bengalensis* from India, Myanmar and Thailand (full data from (Pocock, 1939; Grassman et al., 2005)) and *P. b. borneoensis* (full data from (Rajaratnam, 2000)) indicated that the Indian and Indochinese subspecies was roughly 20% larger than the Malaysian Borneo subspecies. The analysis of summary data from seven independent authors confirmed the larger size of Amur leopard cats (*P. b. euptilurus*) over four other subspecies (*P. b. chinensis*, *P. b. bengalensis*, and *P. b. borneoensis*). Finally, Thailand *P. b. bengalensis* was found to be larger than *P. b. borneoensis*, although, no differences were found between the latter and *P. b. bengalensis* from the Malaysian Peninsula.

Based on both skull and body morphology, there is a clear North–South gradient from larger forms in the Amur region, through Continental China and the Indochinese Peninsula, to the smaller forms in the Sunda Islands. Therefore, latitudinal environmental variation from cold northern regions, through temperate forests and, finally, to warm equatorial forests has a clear parallel with leopard cat subspecies skull and body size variation. Moreover, size differences are also observed when continental and island morphotypes are compared.

*Prionailurus bengalensis* is a relatively common small cat in Southeast Asia. Its wide distribution and adaptation to a broad variety of habitats from tropical rainforest to temperate broadleaf, and marginally coniferous forest and islands (Sunquist & Sunquist, 2002; Rajaratnam et al., 2007) raise important ecological and biogeographical questions. Our original data showed three clusters of specimens (Figs. 1 and 2), structured according to regions in Southeast Asia and suggesting facts related to a common earlier history. Continental China and Hainan Island form a large cluster. Hainan Island is geographically close to mainland China, but geological data show a land connection to Indochina and thence to the Malay Peninsula and the islands of Borneo, Java and Sumatra across the Sunda Shelf, which was exposed during the Last Glacial Maximum *c*. 21,000 years before the present (Sathiamurthy & Voris, 2006; Hanebuth et al., 2011). Skull morphology analyses suggest that the colonization of Hainan Island by the leopard cat was not from the south through the Sunda Shelf. This population must have colonized the island from mainland China, across the Qiongzhou Haixia strait area, as suggested recently by models predicting the distribution of Southeast Asian murids under Last Glacial Maximum conditions (Latinne et al., 2015).

Geographical distance appears to be the primary factor determining relationships. A second cluster of specimens is associated with Indochina, including India and Laos, and includes an individual in an intermediate condition suggesting relations between more tropical regions and mainland China. Data are scarce, but seem geographically structured and warrant further investigation.

The third cluster is composed of specimens from the Sunda Islands of Borneo, Java, and Sumatra. Notably, although the islands (particularly Sumatra) lie close to the Malay Peninsula, the group is characterized as being insular with distinct morphological characteristics. Leopard cats from the Sunda Islands have a narrower and lower-profiled skull, compared to Indochinese forms, suggesting some agreement with evidence for large islands and small species (Meiri et al., 2008). These results diverge from the biogeographical pattern proposed by Leonard et al. (2015), which suggests that several land vertebrates from Sumatra are more closely related to populations on the Malay Peninsula than to those on Borneo. Our data indicate a marked difference between leopard cat morphotypes from Indochina and those from Borneo, Java, and Sumatra, which form a consistent cluster. However, we are dealing with morphometry and not molecular data, as Leonard et al. (2015) were. Notwithstanding, the macaque (*Macaca fascicularis*) is reported by Leonard et al. (2015) as showing a divergent pattern, in which Bornean and Sumatran macaques constitute a clade apart from the Malay Peninsula and mainland forms.

Mammals with wide distributions in Southeast Asia are smaller on Borneo (Meiri et al., 2008), but leopard cats, as an ecologically versatile and widely distributed species in Southeast Asia, may differ less among islands. However, contrasts between mainland specimens (Indochina) and insular specimens are strong, providing further evidence for the role of island resource limitation in size evolution (Foster, 1964; Lomolino, 1985). The patterns between the islands must be further investigated with better databases.

## Conclusions

Leopard cat morphology was analyzed by means of the largest database assembled to date. This database was composed of original data acquired from mammal collections and data published by several authors, in order to deal with the scarcity of material and fragmented information. This study is not a review but, rather, an effort to retrieve and reanalyze information about the morphology and external body measurements of leopard cats assembled over the last 77 years. This study did not include the Iriomote cat due to the scarcity of material, and therefore, unfortunately, we were not able to assess its particular morphology in the context of the leopard cat group. The aim was to unify and systematize all the data and information in order to further the neglected study of morphology and shed some light on the leopard cats’ sexual dimorphism and geographical variation. The outcome was that we demonstrated different degrees of sexual dimorphism in the subspecies. Moreover, several aspects of geographic/subspecific morphological variation were unified in a single study. We took advantage of modern statistical packages to run well-established multivariate tests that were too complex to perform in the first half of the 20th century, when part of this overall data set was collected. The analysis of summary data proved to be a useful tool when full data were not available. However, it does not replace the accuracy of a statistical analysis of large samples with full data and should be used with caution and supplemented with additional analyses. The lack of unified, standard morphometric parameters hindered more comprehensive comparisons between data sets.

A consistent morphological definition of species is the classical first step for thorough biological studies and conservation programs, but sometimes several steps are taken without one. Thus, many studies and subsequent reviews are inconsistent with regard to this basic aspect of species biodiversity. This study aimed to fill this gap by providing a framework for a better understanding of the morphological complexity involved in sexual and geographical variation in leopard cats. We hope that a formal comprehension of the morphological variation present in *P. bengalensis* will provide additional support to the efforts to conserve the species and subspecies.

## Supplemental Information

10.7717/peerj.1309/supp-1 Supplemental Information 1 Specimens measured in the American Museum of Natural History (AMNH), NY, USA

## Additional Information and Declarations

### Data Availability

The following information was supplied regarding data availability: Figshare: http://dx.doi.org/10.6084/m9.figshare.1539651.
