# Supplementary material for: Variations in leopard cat (Prionailurus bengalensis) skull morphology and body size: sexual and geographic influences
Source: PeerJ. 2015 Oct 6;3:e1309. doi: 10.7717/peerj.1309 (PMC4614805; doi:10.7717/peerj.1309)
Supplement: Supplemental Information 1 [file peerj-03-1309-s001.doc]

Supplementary Information

Specimens measured in the American Museum of Natural History (AMNH), NY, USA.

| AMNH 163609 | *Prionailurus bengalensis bengalensis* | ♀ | Chin | Myanmar |
| --- | --- | --- | --- | --- |
| AMNH 87351 | *Prionailurus bengalensis bengalensis* | ♀ | Nam Khan | Vietnam |
| AMNH 87355 | *Prionailurus bengalensis bengalensis* | ♂ | Plateau Bolovens | Laos |
| AMNH 55564 | *Prionailurus bengalensis bengalensis* | n/s | Trang | Thailand |
| AMNH 83999 | *Prionailurus bengalensis chinensis* | ♂ | Fujian | China |
| AMNH 84398 | *Prionailurus bengalensis chinensis* | ♀ | Fujian | China |
| AMNH 57062 | *Prionailurus bengalensis chinensis* | ♀ | Fujian | China |
| AMNH 58371 | *Prionailurus bengalensis chinensis* | ♀ | Sichuan | China |
| AMNH 185464 | *Prionailurus bengalensis chinensis* | n/s | Hong Kong | China |
| AMNH 110458 | *Prionailurus bengalensis chinensis* | n/s | Sichuan | China |
| AMNH 59959 | *Prionailurus bengalensis alleni* | ♀ | Hainan | China |
| AMNH 59957 | *Prionailurus bengalensis alleni* | ♀ | Hainan | China |
| AMNH 60054 | *Prionailurus bengalensis alleni* | ♂ | Hainan | China |
| AMNH 57376 | *Prionailurus bengalensis alleni* | ♂ | Hainan | China |
| AMNH 101628 | *Prionailurus bengalensis javanensis* | ♂ | Cirebon | Java |
| AMNH 102072 | *Prionailurus bengalensis javanensis* | ♀ | Cirebon | Java |
| AMNH 102085 | *Prionailurus bengalensis javanensis* | ♀ | Cirebon | Java |
| AMNH 102073 | *Prionailurus bengalensis javanensis* | ♂ | Cirebon | Java |
| AMNH 102458 | *Prionailurus bengalensis javanensis* | ♂ | Cirebon | Java |
| AMNH 102212 | *Prionailurus bengalensis sumatranus* | ♂ | Lubuklinggau | Sumatra |
| AMNH 104000 | *Prionailurus bengalensis borneoensis* | ♂ | Indeterminada | Borneo |
| AMNH 103709 | *Prionailurus bengalensis borneoensis* | ♂ | Sampit | Borneo |
| AMNH 106063 | *Prionailurus bengalensis borneoensis* | ♂ | Riam | Borneo |
| AMNH 22904 | *Prionailurus bengalensis bengalensis* | ♂ | -- | India |
| AMNH 238413 | *Prionailurus bengalensis* | ♀ | -- | -- |
